# Recent Advances in Endothelial Progenitor Cells Toward Their Use in Clinical Translation

**DOI:** 10.3389/fmed.2018.00354

**Published:** 2018-12-18

**Authors:** Cameron Keighron, Caomhán J. Lyons, Michael Creane, Timothy O'Brien, Aaron Liew

**Affiliations:** Regenerative Medicine Institute, National Centre for Biomedical Engineering Science and Centre for Research in Medical Devices, National University of Ireland, Galway, Ireland

**Keywords:** endothelial colony forming cells (ECFCs), Endothelial progenitor cell (EPC), clinical translation, clinical trial, preclinical study

## Abstract

Since the discovery of Endothelial Progenitor Cells (EPC) by Asahara and colleagues in 1997, an increasing number of preclinical studies have shown that EPC based therapy is feasible, safe, and efficacious in multiple disease states. Subsequently, this has led to several, mainly early phase, clinical trials demonstrating the feasibility and safety profile of EPC therapy, with the suggestion of efficacy in several conditions including ischemic heart disease, pulmonary arterial hypertension and decompensated liver cirrhosis. Despite the use of the common term “EPC,” the characteristics, manufacturing methods and subset of the cell type used in these studies often vary significantly, rendering clinical translation challenging. It has recently been acknowledged that the true EPC is the endothelial colony forming cells (ECFC). The objective of this review was to summarize and critically appraise the registered and published clinical studies using the term “EPC,” which encompasses a heterogeneous cell population, as a therapeutic agent. Furthermore, the preclinical data using ECFC from the PubMed and Web of Science databases were searched and analyzed. We noted that despite the promising effect of ECFC on vascular regeneration, no clinical study has stemmed from these preclinical studies. We showed that there is a lack of information registered on www.clinicaltrials.gov for EPC clinical trials, specifically on cell culture methods. We also highlighted the importance of a detailed definition of the cell type used in EPC clinical trials to facilitate comparisons between trials and better understanding of the potential clinical benefit of EPC based therapy. We concluded our review by discussing the potential and limitations of EPC based therapy in clinical settings.

## Introduction

The term “putative endothelial cell progenitors” was pioneered by Asahara et al in their seminal publication in Science over two decades ago (1). They showed that this cell population can be successfully isolated from peripheral blood derived mononuclear cells of healthy volunteers, utilizing magnetic bead positive selection of two cell surface antigens, CD34, and Flk1. They also demonstrated that EPCs could home specifically to areas of ischemia. This forms the basis of vasculogenesis, whereby new blood vessels are formed by EPCs or angioblasts, which home, differentiate, proliferate, and incorporate into resident mature vessels in response to various stimuli such as ischemia (2). Prior to the discovery of this cell population, the principal mechanism of vascularization after an ischemic event was thought to be due to the process of angiogenesis, whereby new vessels are formed by direct migration, differentiation, and proliferation of the existing mature endothelial cells (3). Furthermore, the discovery of this novel EPC concept has overturned the previous dogma which suggested that vasculogenesis could only occur during embryogenesis. In fact, both vasculogenesis and angiogenesis may potentially have a synergistic role in postnatal revascularization.

The new paradigm shift in the understanding of vascular regeneration has led to multiple publications using the term “EPC.” Specifically, this term has been widely utilized in many studies as a surrogate biomarker to assess the risk of cardiovascular disease in human subjects (4–6) and as a potential novel therapeutic agent for vascular regeneration (7, 8). The initial nomenclature of EPC encompasses a heterogeneous cell population, including early EPC [or circulating angiogenic cells (CAC), myeloid angiogenic cells (MACs), pro-angiogenic haematopoietic cells (PACs)] and late EPC [or outgrowth endothelial cells, endothelial colony forming cells (ECFC)] (9–12).

Early EPCs typically are cultured on fibronectin coated plates and appear early in culture (4–7 days). They are defined as spindled shaped cells with low proliferative potential, with cells only surviving up to 4 weeks in culture (13, 14). Functionally, early EPCs neither form a colony in culture nor vessels *in vitro* in 2D Matrigel assays. They also do not integrate into pre-existing vessels. Despite this, they have been found to have pro-angiogenic paracrine capabilities, demonstrated by their ability to increase the number of tubules formed by mature endothelial cells (13). Medina et al. showed that early EPCs possessed an expression profile more similar to monocytes than endothelial cells, with cells expressing haematopoietic markers (RUNX1,WAS,LYN) as well as inflammatory markers (TLRs,CD14,HLAs) (12). Subsequently, Medina et al. showed that early EPCs were of myeloid origin as opposed to endothelial in origin (10). In contrast, late EPCs are cultured on collagen coated plates and tend to appear later in culture (usually after 1 week) as colonies with well circumscribed monolayers of cells pertaining a cobblestone morphology. Late EPCs behave functionally like endothelial cells with the ability to form vessels in 2D Matrigel angiogenesis assays. They also have an expression profile similar to mature endothelial cells (CD34^+^,VE-Cad^+^,vWF^+^) but have a much higher proliferation rate, and survival, with cells surviving up to 12 weeks. These cells secrete less angiogenic factors compared to early EPCs (9, 10, 12, 14). A more in depth comparison of these cell types can be found in a review by Hirschi et al. (15).

A recent Consensus Statement on Nomenclature of endothelial progenitors has discouraged the current liberal use of the term “EPCs,” and recommends the term “ECFC” instead. They have also proposed a more precise characterization of ECFCs based on a pre-defined cellular phenotype and function (10). This is a crucial step in defining the term “EPC” which will lead to the harmonization and standardization of the cell type used in clinical studies, thus allowing comparisons to be made across different studies.

The objective of this review was to search the current literature and critically appraise the current use of EPCs as therapeutic agents by summarizing: (a) clinical trials using EPCs as a therapeutic agent currently registered in www.clinicaltrials.gov; (b) published clinical trials using EPCs as a therapeutic agent; and (c) the efficacy of ECFCs in preclinical and clinical studies. We also highlight the potential and limitations of EPC based therapy in clinical settings.

## Materials and Methods

Three separate literature searches were conducted to generate the data presented here (Appendices 1–3). “EPC” was used as the first search term and this was carried out using the www.clinicaltrials.gov database, focusing specifically on interventional therapies only. The second literature search focused on clinical trials in humans using the term “EPCs” as a therapeutic intervention using the PubMed and Web of Science databases published within the last 10 years. The third literature search (PubMed and Web of Science databases) was performed using the synonymous names of ECFCs, “Endothelial Colony Forming Cells or Outgrowth Endothelial Cells or Blood Outgrowth Endothelial Cells or Endothelial Outgrowth Cells or Late Endothelial Progenitor Cells or Late Outgrowth Endothelial Progenitor Cells,” as defined by Medina et al. (10) as the search term. Results were limited to primary interventional studies carried out within the last 10 years.

## Results

### Clinical Trials Using EPCs As Therapeutic Agent, Registered Under www.clinicaltrials.gov Registry

A total of 341 clinical trials were found when the term “EPC” was searched using the www.clinicaltrials.gov registry (22/09/05 to 12/04/17). Many of the registered trials related to the assessment of EPCs across various clinical states and were non-interventional in nature. There are 26 clinical trials which utilized EPCs as a therapeutic agent currently registered in www.clinicaltrials.gov (Table 1) with a total of 1,148 participants expected to be enrolled into the trials. Approximately two thirds of these registered clinical trials involved patients with ischemic conditions such as peripheral artery disease (*n* = 8), coronary artery disease (*n* = 7) and ischemic stroke (*n* = 2). The remaining conditions registered were pulmonary arterial hypertension (*n* = 4), liver cirrhosis (*n* = 2), lymphodema (*n* = 1), erectile dysfunction (*n* = 1), and traumatic bone defects (*n* = 1) (Table 1).

**Table 1:** Clinical trials using EPCs as therapeutic agent, registered under www.clinicaltrials.gov registry (22^nd^ Sept 2005–12^th^ Apr 2017).

**NCT**	**Condition**	**Phase**	**n**	**Cell type**	**Cell surface marker(s)**	**Cell culture condition**	**Status**	**Sponsor(s)**	**Published results**
NCT00936819	Acute Myocardial Infarction	II	100	PB-EPCs or eNOS-EPCs	CD31, CD34, CD14, CXCR4, VEGFR2	2-3 days culture in fibronectin coated plates	Recruiting	Ottawa Hospital Research Institute	NR
NCT01049867	Coronary Artery Disease	I/II	10	BM-EPCs	CD133^+^	NR	Unknown	Hospital y Clinica OCA, S.A. de C.V.	NR
NCT00384514	Coronary Artery Disease	II	24	PB-EPCs	NR	NR	Unknown	TheraVitae Ltd	NR
NCT00289822	Coronary Artery Disease	II	75	PB-EPCs	NR	0 or 3 days culture	Terminated	Johann Wolfgang Goethe University Hospital	De Rosa et al. (16)
NCT00629096	Dilated Cardiomyopathy	II	27	BM-MNCs	NR	NR	Completed	Fundación Pública Andaluza Progreso y Salud	NR
NCT00221182	Heart Disease	I/II	1	PB-EPCs	CD34^+^	NR	Terminated	Foundation for Biomedical Research and Innovation	NR
NCT00694642	Refractory Angina	I/II	28	EPCs	CD133^+^	N/A	Completed	Pilar Jimenez Quevedo	Jimenez-Quevedo et al. (17)
NCT02605707	Chronic Ischemic Stroke	I/II	30	EPCs	NR	NR	Unknown	Southern Medical University, China	NR
NCT01468064	Ischemic Stroke	I/II	20	BM-EPCs	NR	NR	Unknown	Southern Medical University, China	NR
NCT01595776	Critical Limb Ischemia	I/II	8	PB-EPCs	CD133^+^	NR	Completed	IRCCS Policlinico S. Mattteo	Arici et al. (18)
NCT02454231	Critical Limb Ischemia	II/III	45	PB-EPCs	CD14^+^, CD34^+^	NR	Completed	University of Florence	NR
NCT00523731	Critical Limb Ischemia	I	6	PB-EPCs	NR	Cells cultured in X-vivo 15 serum free medium supplemented with autologous human serum, VEGF and heparin for 5 days	Completed	TheraVitae Ltd	Mutirangura et al. (19)
NCT02287974	Critical Limb Ischemia	I/II	20	BM-EPCs	CD133^+^	NR	Active	Andalusian Initiative for Advanced Therapies - Fundación Pública Andaluza Progreso y Salud	NR
NCT02474381	Diabetic Foot	NR	60	EPCs	CD133^+^	NR	Unknown	Shanghai 10th People's Hospital	Zhang et al. (20)
NCT00221143	No Option Critical Limb Ischemia	I/II	15	PB-EPCs	CD34^+^	NR	Completed	Translational Research Informatics Centre, Kobe, Hyogo, Japan	Kawamoto et al. (21), Kinoshita et al. (22)
NCT02915796	Peripheral Arterial Disease	I	345	PB-EPCs	CD133^+^	Mononuclear cells isolated, re-suspended in RPMI-1640 and then injected into the ischemic area	Recruiting	Shanghai 10th People's Hospital	Huang et al. (23)
NCT00306085	Peripheral Atherosclerosis	I	20	BM-Cells	CD34^+^	Cells sorted by the MarrowXpress system and immediately transplanted	Unknown	University of Naples	Malone et al. (24), Cobellis et al. (25)
NCT00641836	Idiopathic PAH	NR	98	PB-EPCs	VE-Cad^+^, KDR^+^, CD34^+^, AC133^+^	Cultured on fibronectin coated flasks in Medium 199 for 5 days	Completed	Zhejiang University	NR
NCT00257413	Idiopathic PAH	NR	31	PB-EPCs	VE-Cad^+^, KDR^+^, CD34^+^, AC133^+^	Cultured on fibronectin coated flasks in Medium 199 for 5 days	Completed	Zhejiang University	Wang et al. (26)
NCT00372346	Idiopathic PAH	NR	40	PB-EPCs	vWF^+^, CD31^+^, CD34^+^	Cultured on fibronectin coated flasks in EGM-2 for 10-14 days	Unknown	Zhejiang University	NR
NCT00469027	PAH	I	7	PB-eNOS-EPCs	CD14^+^, CD31^+^	Isolated and cultured on fibronectin coated flasks for 7-12 days	Completed	Northern Therapeutics	Granton et al. (27)
NCT01333228	Liver Cirrhosis	I/II	14	BM-EPCs	CD31, CD34, CD14, VEGFR-2, VEGFR-1, CD133, CD90, CD117, vWF, CXCR1, CD45, ID1	BM-EPCs isolated via Ficoll gradient, cultured on fibronectin coated plates in endothelial complete medium for 4 days	Completed	Clinica Universidad de Navarra + Universidad de Navarra	D'Avola et al. (28)
NCT03109236	Liver Cirrhosis	III	66	BM-EPCs	CD133^+^	NR	Recruiting	National University Hospital, Singapore	NR
NCT01112189	Lymphedema	I/II	20	BM-EPCs	NR	NR	Completed	Hospital Universitario Dr. Jose E. Gonzalez	Maldonado et al. (29)
NCT01089387	Post Prostatectomy Erectile Dysfunction	I/II	18	BM-MNCs	NR	NR	Completed	Institut National de la Santé Et de la Recherche Médicale, France	NR
NCT03103295	Traumatic Bone Defects	I/II	20	PB-EPCs	NR	Cultured in EGM	Active	A.A. Partners, LLC	Vasyliev et al. (30)

*BM, Bone Marrow; eNOS, Endothelial Nitric Oxide Synthase; EPCs, Endothelial Progenitor Cells; MNC, Mononuclear Cells; N/A, Not Applicable; NR, Not Reported; PAH, Pulmonary Arterial Hypertension; PB, Peripheral Blood*.

The majority of the 26 registered clinical trials specified the source for EPC in the registry (*n* = 19), such as bone marrow derived (*n* = 9) or peripheral blood derived (*n* = 10), but only 10 trials specified the cell surface markers used for EPC definition in the registry, including CD133^+^(*n* = 7), CD34^+^(*n* = 2), and CD14^−^(*n* = 1). The status of half of these trials were labeled as “complete” (*n* = 12). Two trials were labeled as “terminated.” Slow recruitment was noted for one of these two trials (NCT00221182). The other trial was subsequently published (NCT00289822) (16). The remaining trials were labeled as “active” (*n* = 2), “recruiting” (*n* = 3), and “unknown” (*n* = 7).

The results of this search showed that the term “EPC” has been widely used to encompass a heterogeneous population of cells. In addition, these results also highlight the paucity of information registered within the www.clinicaltrials.gov for these clinical trials, thereby preventing direct comparison with other trials. A detailed definition of the cell type used in clinical trials is warranted, to facilitate better understanding of the potential clinical benefit of EPC based therapy.

### Published Clinical Trials Using EPCs As the Therapeutic Agent

Five relevant published clinical trials using EPCs were found following the PubMed and Web of Science database searches (01/05/2008 to 01/05/2018). These clinical trials involved various disease states including peripheral arterial disease (31, 32), coronary artery disease (33), pulmonary hypertension (34), and liver cirrhosis (28) (Supplementary Table 1). Despite using the term “EPC” as a therapeutic agent, the cell types used in all the five published trials differ, specifically, in their methods of cell isolation and culture, as well as the cell surface markers used for phenotypic characterization. Furthermore, the cell dose, and the route of administration varies between trials.

Lara-Hernandez et al. showed that intramuscular administration of EPCs into the ischemic limbs of 28 patients with no-option critical limb ischemia was safe and feasible (31). They used 50 mls of G-CSF mobilized blood and then selected for CD34^+^ and CD133^+^ cells. This treatment resulted in a significant reduction in the pain score of no option CLI patients with increased tissue perfusion and no adverse effects noted after a follow-up of 14 months (31). Tanaka et al. also isolated EPCs from G-CSF mobilized blood, but selected for CD34^+^ EPCs prior to intramuscular administration into non-healing diabetic feet. They demonstrated the safety of EPC therapy, no serious adverse events noted, coupled with increased vascular perfusion and complete wound closure after ~18 weeks (32).

Zhu et al. demonstrated the safety and feasibility of EPCs by injecting thymosin β1 pre-treated EPCs, into patients with ST segment elevated myocardial infarction, with some efficacy data showing improved exercise capacity and left ventricular function after 6 month follow-up (33). The EPCs used by Zhu et al. were cultured on fibronectin and phenotypically characterized as VE-Cadherin^+^, KDR^+^, CD34^+^, and CD133^+^ (33) cells. Similarly, Zhu et al demonstrated the safety and feasibility of intravenously administered CD34^+^, CD133^+^, and KDR^+^ EPCs into children with idiopathic pulmonary arterial hypertension (IPAH). Although safety was their primary endpoint, significant increases in exercise capacity and pulmonary hemodynamics were also noted (34).

D'Avola et al. showed that a single administration of EPCs (maximum of 100 × 10^6^ cells) via the hepatic artery in 12 patients with decompensated liver cirrhosis is safe and feasible (28). No treatment-related severe adverse events were seen for up to 1 year of follow up. Extensive characterization of EPCs was performed following 7 days of *ex vivo* culture of fibronectin adherent mononuclear cells (from 50-100 mls of bone marrow aspirate) using extensive cell surface markers (CD31, CD34, CD14, VEGFR2, VEGFR1, CD133, CD90, CD117, vWF, CXCR4, ID1, and CD45) and functionality assessment with DiL-acetylated-LDL cholesterol uptake and lectin binding capacity as well as demonstration of tube-structure formation over Matrigel matrix (28).

While some of the clinical data from Table 1 was published, several of these papers were not included in this section for overall analysis for the following reasons: one paper did not use the term EPC but instead used the term “angiogenic precursor cells” (19), five papers included cells identified only using a single cell surface marker [e.g., CD133^+^ (17, 18, 20) or CD34^+^ (21, 29)], and one did not specifically define the cell type (24). It was concluded that these papers could not be classified according to our search criteria as being interventional EPC studies, and thus excluded. Two papers were published beyond the date range specified in our search (23, 26). Finally, two other papers from Table 1 met our classification criteria did not appear in our literature search (27, 30). Granton et al. used 7–50 × 10^6^ peripheral blood eNOS modified EPCs for pulmonary arterial hypertension and showed an improvement in pulmonary resistance after treatment (27), whereas Vasyliev et al. used 20–60 × 10^6^ autologous peripheral blood derived EPCs in a scaffold of allogeneic bone and fibrin gel for bone fracture repair and demonstrated an improvement in bone regeneration (30). Of note, neither study had a control group to compare against.

Overall, all trials were single arm trials, with four of the five published trials being early phase clinical trials and demonstrated safety and feasibility of EPC therapy. Only Zhu et al. conducted a randomized controlled trial (33).

### Efficacy Data Using ECFCs

In the previous sections, we examined human interventional studies using “EPCs” as a therapeutic but had noted that the field is moving toward a cell type that is considered to be the “true EPC.” Thus, we examined the published data on ECFCs, the novel and “bone-fide” EPC, using the PubMed and Web of Science databases. From our search using all the relevant search terms, only 39 out of 1,316 papers retrieved used hECFCs as an interventional therapy in preclinical models (01/05/2008 to 01/05/2018) (Table 2). The majority of the published articles assessed the potential therapeutic role of ECFC in ischemic disease models including hindlimb ischemia (*n* = 20), cerebral ischemia (*n* = 4), retinal ischemia (*n* = 2), myocardial ischemia (*n* = 2), ischemic acute kidney injury (*n* = 1) and ischemic reperfusion injury (*n* = 1). However, ECFCs were also being investigated for other conditions such as islet graft retention (*n* = 3), vascular injury (*n* = 2), pulmonary arterial hypertension (*n* = 1), bronchopulmonary dysplasia (*n* = 1) and traumatic brain injury (*n* = 2). In these 39 papers, hECFCs were predominantly derived from the umbilical cord (*n* = 31), followed by peripheral blood (*n* = 6) (Table 2). However, despite a robust preclinical evidence to suggest the efficacy of ECFCs in several conditions, this cell type has yet to be tested in human clinical trials.

**Table 2:** Preclinical work using ECFCs as a therapeutic agent.

**Condition**	**Cell source**	**Cell number**	**Cell surface marker(s)**	**Administration method**	**Recipient**	**n**	**Outcome**	**References**
Hind Limb Ischemia	hUC-ECFCs or EPO Primed hUC-ECFCs	1 × 10^5^	CD131^+^, EPOR^+^	IV	Athymic Nude Mice	5–7	Primed ECFCs have improved graft efficiency, improved cell survival and improved angiogenic potential	Bennis et al. (35)
Hind Limb Ischemia	hUC-ECFCs	1 × 10^5^	CD34^+^, CD31^+^, Tie-2^+^, KDR^+^, Flt-1^+^, CD144^+^, CD14^−^, CD45^−^	IV	Athymic Nude Mice	10	Improved residual muscle blood flow and increased collateral vessel formation	Sarlon et al. (36)
Hind Limb Ischemia	hUC-ECFCs	1 × 10^6^	CD34, CD146, CD45, KDR	IM injection to 3 sites (In 10 mice VEGF was blocked)	C57BL/6 N mice	50	ECFC treated mice showed significantly better outcomes in recovery quality and length	Flex et al. (37)
Hind Limb Ischemia	hUC-ECFCs	1 × 10^5^	CD34^+^, CD31^+^, Tie-2^+^, KDR^+^, Flt-1^+^, CD144^+^, CD14^−^, CD45^−^	IM injection to ischemic area	Athymic nude mice	6	Improved blood flow	Mena et al. (38)
Hind Limb Ischemia	hUC-ECFCs	1 × 10^6^	NR	Injection into three sites (20 μl/each site) of the gracilis muscle in the medial thigh three times/ week	Male C57BL/6J mice	8	Improved blood flow	Kim et al. (39)
Hind Limb Ischemia	hUC-ECFCs	1 × 10^5^ cells dissolved in 500 μl of PBS	CD34+	IV	NOD/Shi-scid, IL-2Rγnull mice	15	Improved blood flow	Goto et al. (40)
Hind Limb Ischemia	hUC-ECFCs	(i) CAC-CM (50 μl) (ii) ECFC-CM (50 μl) (iii) ECFC (2 × 10^5^ cells/50 μl), (iv) CAC (10^6^ cells/50 μl), (v) a mix containing CAC-CM (25 μl) and ECFC suspension (10^5^cells/25 μl), or (vi) a mix containing CAC suspension (5 × 10^5^cells/25 μl) and ECFC-CM (25 μl)	CD31^+^, CD144^+^, KDR^+^, VEGF^+^, Flk-1^+^, CD14^−^, CD45^−^	Matrigel implantation into ischemic site	C57BL/6 N mice	3	Endothelial cell retention and vascular maturation	Odent Grigorescu et al. (41)
Hind Limb Ischemia	hUC-ECFCs	5 × 10^5^ cells (IM) or 1 × 10^6^ cells (IV)	CD34^+^, vWF^+^, CD133^+^, KDR^+^, CD31^+^, c-kit^+^, CXCR4^+^, CD144^+^, eNOS^+^, p-eNOS^+^, VEGFR2^+^	IM or IV injection	Balb/C Nude Mice	5	Significantly enhanced blood perfusion, capillary density, proliferation and angiogenic cytokine secretion	Lee et al. (42)
Hind Limb Ischemia	hUC/PB-ECFCs & hMSCs	NR	CD31^+^, KDR^+^, CD34^+^	IV	Nude Mice	8–10	Enhanced neovascularization	Schwarz et al. (43)
Hind Limb Ischemia	hUC-ECFC	1 × 10^5^	CD34^+^	IV	Type 2 diabetic C56BL/6 J male athymic Nude mice	6	Increased blood flow recovery and vascular density, with reduced inflammation	Mena et al. (44)
Hind Limb Ischemia	Egfl7 repressed hUC-ECFCs	1 × 10^5^	CD31^+^, CD34^+^, CD144^+^, CD133^−^, CD45^−^, CD90^−^	IV	Athymic nude mice	14	Improved revascularisation	D'Audigier et al. (45)
Hind Limb Ischemia	hUC-ECFCs treated with epigenetic drugs (GSK-343 and panobinostat)	5 × 10^5^	CD31^+^, CD34^+^, CD45^−^	IM	NOD/SCID and athymic nude CD1 female mice	7–8	Increased vasculogenesis	Fraineau et al. (46)
Hind Limb Ischemia	hBM-MSC conditioned medium + hUC-ECFCs	1 × 10^5^	CD34^+^, CD144^+^, CD146^+^, KDR^+^, CD45^−^, CD14^−^	IV	NMRI-nude mice	6	Increased blood perfusion	Poitevin et al. (47)
Hind Limb Ischemia	Trichostatin A treated hUC-ECFCs	5 × 10^5^	CD34^+^, CD31^+^, CD105^+^, CD144^+^, VEGFR2^+^, vWF^+^, CD45^−^, CD14^−^	IM	Athymic nude CD1 female mice	3–4	Enhanced vascular repair capacity	Palii et al. (48)
Hind Limb Ischemia	hUC-ECFCs	1 × 10^6^	CD31^+^, Flk^+^, vWF^+^, eNOS^+^, phospho-eNOS^+^	IV	Male C57BL/6J or BALB/c-nu/nu mice	8	Improved neovascularization and limb salvage	Heo et al. (49)
Hind Limb Ischemia	α6 knockdown hUC-ECFCs	1 × 10^5^	CD31^+^, CD34^+^, CD144^+^, CD146^+^, CD45^−^, CD14^−^	IV	Male athymic nuce Foxn-1 mice	5	No ECFC integration or neovascularization	Bouvard et al. (50)
Hind Limb Ischemia	BMP2 or BMP4 treated hUC-ECFCs + hPB-ECFC	NR	VEGFR2^+^, CD31^+^, CD34^+^, CD45^−^, CD14^−^	IV	Nude mice	NR	Increased therapeutic potential of ECFCs exposed to BMP	Smadja et al. (51)
Hind Limb Ischemia	PB-ECFCs derived from white European and south Asian males	3 × 10^5^	CD31^+^, CD144^+^, CD146^+^, CD309^+^, CD45^−^, CD14^−^	IV	Male immunodefic-ient CD1 nude mice	5	Superior recovery in ECFCs from white European compared to those from South Asian men	Cubbon et al. (52)
Hind Limb Ischemia	Non-diabetic controls (young + age matched) + type 2 diabetic hPB-ECFCs treated with globular adiponectin	5 × 10^5^	CD34^+^, CD31^+^, VEGFR2^+^	IV	Diabetic female athymic NMRI nu/nu mice	4–8	Increased and prolonged neovascularization in adiponectin treated diabetic ECFCs compared to untreated diabetic ECFCs	Leight et al. (53)
Hind Limb Ischemia	ECFCs + hBM-MSCs	NR (1:1 ratio)	NR	Retro-orbital injection	Athymic male nude mice	6–7	Significantly higher vessel perfusion in ECFC only group, and significantly higher density and foot perfusion after co-transplantation	Rossi et al. (54)
Ischemic retina	hUC-ECFCs	1 × 10^3^, 1 × 10^4^, 1 × 10^5^	CD31^+^, CD105^+^, CD14^−^, and CD45^−^	Intravitreal Delivery	P13 mice	1–8	Low dose cohort showed best improvement	Reid et al. (8)
Ischemic retina	Low passage and late passage hPB-ECFCs + hUC-ECFCs	NR	VEGFR2^+^, Caveolin 1^+^,CD45^−^, CD14^−^, CD31^+^, CD105^+^, CD146^+^, CD34^+^	Intravitreal injection	C57BL/6 mice	6	Late passage ECFCs had impaired vasoreparative properties	Medina et al. (55)
Ischemic Myocardium	OECs	5 × 10^6^	CD45^−^, CD133^+^	Intramyocardial injection	Rabbits	8	Improved cardiac function	Tan et al. (56)
Myocardial Infarction	hUC-ECFCs	5 × 10^6^	CD31^+^, CD34^+^, CD105^+^, CD144^+^, CD146^=^, KDR, Tie-2^+^, CD45^−^	Intramyocardial injection	Male Sprague-Dawley rats	5	Increased angiogenesis and improved cardiac function	Kim et al. (57)
Ischemia Reperfusion Injury	hUC-ECFC + MPCs	2 × 10^6^ (2:3)	CD31^+^	Intracoronary injection	Nude Rats	3–17	Higher LV dimensions, higher heart weight to tibia length ratio. Improved cardiac function	Kang et al. (58)
Ischemic AKI	hUC-ECFCs	1 × 10^6^	CD31^+^, VEGFR2^+^, CD45^−^, CD14^−^, CD133^−^	IV	Male NOD-SCID (NOD.CB17-*Prkdc^*scid*^*/J)	5-7	ECFCs protect against ischemic AKI damage	Burger et al. (59)
Vascular Injury	MSC derived ECFCs	5 × 10^5^	CD133^+^ CD34^+^ KDR^+^, vWF^+^, CD31^−^, CD45^−^	IV through tail vein	Male nude mice	120	Accelerated re-endotheliazation and inhibits neointimal hyperplasia	Wang et al. (60)
Vascular Injury	hPB-ECFCs pretreated with recombinant BMP4	5 × 10^5^	CD31^+^, KDR^+^, Tie-2^+^	IV	Male NRMInu/nu athymic nude mice	5	Accelerated endothelial repair capacity	Xia et al. (61)
Traumatic Brain injury	hUC-ECFCs	3 × 10^5^	CD34^+^, KDR^+^, vWF^+^, VE-Cad^+^, UEA-1^+^	Intra-cerebroventricular Infusion	Balb/C Nude Mice	36	Reduced Evans blue extravasation, reduced brain water content. Increased microvascular density. Improved neurological function	Huang et al. (62)
Traumatic Brain injury	hUC-ECFC	1 × 10^6^	CD31^+^, vWF^+^, VE-Cad^+^	IV	Balb/C Nude Mice	21	Improved rate of neurologic disability. Increased microvessel density and proangiogenic growth factors SDF-1 + VEGF	Zhang et al. (63)
Cerebral Ischemia	hUC-ECFCs	4 × 10^6^	CD146^+^	IV via tail vein	Adult male Sprague–Dawley rats	33	Erythropoietin primed ECFCs showed best improvement	Garrigue et al. (64)
Transient Focal Cerebral Ischemia	hUC-ECFCs + EPO	5 × 10^6^	NR	IV	Sprague-Dawley Rats	24	Completely restored neurological function	Pellegrini et al. (65)
Stroke (Middle Cerebral Artery Occlusion)	hUC derived ECFCs	4 × 10^6^	CD54^+^, CD31^+^, CD146^+^, CD34^+^, CD144^+^, KDR^+^ CD45^−^, CD14^−^, CD133^−^	IV via femoral vein	Adult male Sprague-Dawley rats	4–37	Improved functionality	Moubarik et al. (66)
Ischemic Stroke	hUC-ECFCs	1 × 10^6^	CD31^+^, CD34^+^, VEGFR2^+^, CD133^−^	Injected into the left ventricle	Male BALB/c-nu mice	NR	Functional recovery, improved angiogenesis + neurogenesis with reduced apoptosis	Ding et al. (67)
Islet Graft Retention	hPB-ECFC	5 × 10^5^	NR	Infra-Kidney Transplantation	Hypergylcaemic NOD-SCID Mice	6-9	Improved β-cell survival and graft-vessel and β-cell volume	Coppens et al. (68)
Islet Graft Retention	hUC-ECFCs	6 × 10^5^	CD31^+^, VE-Cad^+^, CD105^+^, vWF^+^, KDR^+^	Infra-Kidney Islet Transplantation	Balb/C Nude Mice	6-7	Absence of blood inflammatory reaction	Kim et al. (69)
Islet Graft Retention	hUC-ECFCs	NR	VE-Cad^+^, KDR^+^, Flt-1^+^, eNOS^+^, vWF^+^, CD31^+^	Intraportal Islet Transplantation	Diabetic Balb/C Nude Mice	23	Improved rate of neurologic disability. Increased microvessel density and proangiogenic growth factors SDF-1 + VEGF	Jung et al. (70)
PAH	hPB-ECFCs and hPB-EPCs	1.5 × 10^6^	CD31^+^, KDR^+^, CD14^−^, CD34^+^	IV	Male nude rats	4–22	ECFCs had poor retention and no efficacy. EPCs resulted in right ventricular hypertrophy and increased right ventricular systolic pressure	Ormiston et al. (71)
BD	hUC-ECFCs	1 × 10^5^ into mice, 2.5 × 10^5^ into rats	CD31^+^, CD105^+^, CD144^+^, CD146^+^, CD14^−^, CD45^−^	IV	Rag–/– mice and RNU nude rats	5	No adverse effects with improvements in lung structure, exercise capacity and pulmonary hypertension	Alphonse et al. (72)

*AKI, Acute Kidney Injury; BD, Bronchopulmonary Dysplasia; BM, Bone marrow; BOEC, Blood Outgrowth Endothelial Cells; CACs, Circulating Angiogenic Cells; CM, Conditioned Media; IM, Intramuscular; IV, Intravenous; KDR, Kinase Insert Domain Receptor; LV, Left Ventricular; MPCs, Mesenchymal Progenitor Cells; m/r/pECFCs, Murine/Rabbit/Porcine Endothelial Colony Forming Cells; MSCs, Mesenchymal Stem Cells; NR = Note reported; OECs, Outgrowth Endothelial Cells; PAEC, Pulmonary Arterial Endothelial cells; PAH, Pulmonary Arterial Hypertension; PB, Peripheral Blood; PMVEC, Pulmonary microvascular endothelial cells; SDF-1, Stromal Cell-Derived Factor-1; UC, Umbilical Cord; vWF, VonWillebrands Factor; VE-Cad, Vascular Endothelial Cadherin; VEGFR2, Vascular Endothelial Growth Factor Receptor 2*.

Medina et al. highlighted the importance of ECFC identification with more than one surface marker (10). Despite the use of the term “ECFC,” four preclinical studies did not confirm the identity of ECFCs using surface expression markers, with eight studies utilizing only one surface expression marker. Of the papers which used multiple markers, the most commonly used surface markers were: CD34^+^ (*n* = 11), CD31^+^ (*n* = 16), VEGFR2/KDR^+^ (*n* = 13), and CD45^−^ (*n* = 14).

## Discussion

The results of our literature search showed that the term “EPC” has been widely used to encompass a heterogeneous population of cells in both the published and registered clinical studies, thereby, rendering direct comparison amongst studies impossible. Our findings also highlight the paucity of information registered within www.clinicaltrials.gov for these clinical trials, specifically, the cell phenotype that is being tested. Approximately half of the EPC clinical trials (11 out of 26 trials) did not define the culture conditions used for their therapeutic product, and a majority of the trials (17 out of 26 trials) did not specify the cell surface markers used to characterize the cell therapy product used in their trials. Whilst the results from many of the ongoing clinical trials are yet to be published/released, only 12 out of 26 trials have reached the completion phase.

From the published EPC human clinical trials, there is a marked inconsistency in terms of the culture conditions and characterization of EPCs with D'Avola et al. and Vasyliev et al using multiple different cell surface markers to identify the EPCs they used (28, 30), Lara-Hernandez et al. and Granton et al using two cell surface markers (27, 31), Tanaka et al. only utilizing one cell surface marker (32), and Zhu et al. and Zhu et al. did not mention the marker used for defining the EPCs in their studies (33, 34). This lack of standardization makes it difficult to compare the results of different clinical trials and will in the future hinder the translation of these therapies into medical practice. A similar lack of consistency is again observed with the methods to culture EPCs with some reports administering EPCs without prior culture (31, 32), some culturing on fibronectin before re-injection into the patient (27, 28, 33, 34), and Vaysilev et al. culturing cells on uncoated flasks with EGM-2 medium (30). Despite the positive results from the above papers, EPC therapy is still limited by the immunogenicity of allogeneic EPCs, along with its poor definition, isolation and expansion standardization, as outlined above.

From the above data it is clear that a detailed definition of the cell type used in clinical trials is warranted, to facilitate better understanding of the potential clinical benefit of EPC based therapy. Consistent with the recommended nomenclature by Medina et al. the term “ECFC,” a more accurate description of a specific cell type within the EPC population can be carried out, allowing the standardization of cell definition for potential therapeutic use (10).

In fact, currently, there are an increasing number of preclinical studies which have demonstrated the efficacy of ECFCs in various disease models (36, 40, 56, 59, 61, 65, 68, 73). These studies predominantly focus on ischemic conditions such as limb ischemia, cerebral ischemia, myocardial ischemia, ischemic reperfusion injury, and ischemic kidney injury. The likely reason for this is beyond the scope of this review article but it may be related to the angiogenic effect of these cells, which facilitate the revascularization in these ischemic states; however these cells may have other unexplained therapeutic effects, which may not be related to its angiogenic properties. However, similar to EPC studies, there is a lack of standardization of cell surface markers and culture protocols when producing ECFCs for therapeutic intervention between the different studies. This lack of standardization, while addressed by Medina et al. (10), has not yet been fully adopted by the field of vascular regeneration.

The ability to successfully isolate ECFCs is crucial prior to its consideration for clinical use. We and others have previously published methods for ECFC isolation (74–78). However, the yield of ECFCs varies depending on the method used (9). Most methods used collagen as the matrix molecule for cell seeding, rather than fibronectin, suggesting that the former molecule is a better cell selection method (74–78). Direct comparison between collagen and fibronectin by Colombo et al. showed the contrasting impact of the type of matrix molecule used for cell seeding on the pharmacodynamics of ECFC colonies. Seeding cells on fibronectin, as compared with collagen, resulted in earlier appearance of ECFC colonies. In contrast, ECFC colonies cultured on collagen demonstrated a better cell proliferation and lifespan, which might be IL-6 and IL-8 dependent (79). Interestingly, the immunophenotype and the ability for *in vitro* tubule formation remains similar despite the type of matrix molecule used for cell seeding (79).

Tasev et al. have further refined the ECFC isolation method with better cell expansion rates using platelet lysate supplemented culture medium, for large scale propagation for potential clinical use (80). Hofmann et al. have also described an easily applicable method for isolating ECFCs directly using adult human blood to generate more than 100 million functional ECFCs (77). They collected 5 ml of peripheral blood from patients and plated directly into a T75 flask discarding supernatants at various time points to remove any blood cells that were not ECFCs. Their method used human platelet lysate, thereby making it a xeno-free protocol, and they were able to consistently isolate and expand ECFCs up to 30 population doublings (81). Moreover, using the culture method outlined in their paper, ECFCs can be cryopreserved without resulting in genomic instability or changes in cell phenotype and function (81).

Siegel et al. have successfully produced an ECFC product by leukapheresis of peripheral blood in accordance with Good Manufacturing Products (GMP) standard (82). Their isolation method can produce approximately 1.44 × 10^8^ ECFC per white blood cell, following leukaparesis of up to 6.8 liters of peripheral blood. These ECFCs showed a significant Dil-AcLDL uptake and showed CD29^+^, CD31^+^, CD34^+^, CD44^+^, CD105^+^, CD117^+^, CD133^+^, CD144^+^, CD146^+^, and VEGFR2^+^ expression. Furthermore, they showed that their ECFCs could reach up to twelve cumulative population doublings. More importantly, these ECFCs showed no evidence of telomerase activity, as well as capable of *in vitro* tubule formation and secretion of epidermal growth factor, HGF, VEGF-A, platelet derived growth factor-B, IL-8, and monocyte chemoattractant protein-1 (82).

The major limitations of ECFC therapy are the long culture times to generate a therapeutic dose, due to its low frequency in peripheral and cord blood, and that it can only be administered in an autologous fashion due to its inherent immunogenicity. Furthermore, despite the potential for ECFC cryopreservation for future use, the intrinsic function of autologous ECFC may be impaired due to the underlying diseased state such as diabetes mellitus, with Jarajapu et al. reporting to be able to isolate ECFC from three in every ten diabetic patients, compared to eight out of nine in non-diabetic controls (83). However, ECFCs can be genetically modified to augment their function *in vivo* which may facilitate correction of disease-induced cell dysfunction. Examples of genetic modification include β1 integrin overexpression to improve blood perfusion in CLI (40), erythropoietin overexpression to promote erythropoiesis (84), or GSK-3β inhibition which improves the angiogenic capabilities of ECFCs (85).

In addition to genetic modification, ECFCs can be combined with other cell types to improve a specific aspect of ECFC therapy, such as combining them with mesenchymal stem cells (MSCs) or mesenchymal progenitor cells to reduce the immunogenic effect of allogeneic ECFCs and to increase cell survival post transplantation (86). This is due to the anti-inflammatory effects of mesenchymal stem cells. Reports have also noted that MSCs can differentiate into pericyte like cells which act to stabilize the vasculature formed (86–90). Based on the results of the above studies, next generation vascular cell therapies will likely consist of genetically modified ECFCs or ECFC combination.

## Conclusions

To date, EPC based therapy has been shown to be feasible and safe with suggestion of efficacy. However, it is important to note that only one trial included a control arm. While EPCs have been previously the favored cell type utilized for vascular therapeutics, they consist of a heterogeneous population of cells which will produce challenges in terms of GMP compliant cell manufacturing and definition of a cell product. “ECFCs,” as defined by Medina et al. have several advantages over EPCs as a therapeutic product, as outlined above, including being a more defined cell type with enhanced proliferation, and possessing the ability to form new vessels, while also integrating into pre-existing vasculature. The use of ECFCs facilitates the harmonization and standardization of the cell type used in clinical studies, allowing direct comparison between studies (10). To translate ECFCs into routine clinical practice, issues surrounding their immunogenicity will need to be overcome, along with the issues regarding the standardization of markers used to identify them. To date, there have been no clinical trials using this cell type.

## Author Contributions

CK, TO, and AL: conception and design of the study. CK, CL, MC, and AL: analysis and interpretation of data. CK, CL, and AL: drafting of the manuscript. All authors revised the manuscript critically for important intellectual content and final approval for the submission of the manuscript.

### Conflict of Interest Statement

TB is a founder, director and equity holder in Orbsen Therapeutics Ltd. TB and AL are both authors on a patent entitled ‘Osteopontin for the prediction and treatment of cardiovascular diseases' (US Patent Number: US8323968B2). The remaining authors declare that the research was conducted in the absence of any commercial or financial relationships that could be construed as a potential conflict of interest.

## References

[1] Asahara T Murohara T Sullivan A Silver M van der Zee R Li T. Isolation of putative progenitor endothelial cells for angiogenesis. Science (1997) 275:964–7. 10.1126/science.275.5302.964 9020076

[2] Asahara T Masuda H Takahashi T Kalka C Pastore C Silver M. Bone marrow origin of endothelial progenitor cells responsible for postnatal vasculogenesis in physiological and pathological neovascularization. Circ Res. (1999) 85:221–8. 10.1161/01.RES.85.3.221 10436164

[3] Risau W Sariola H Zerwes HG Sasse J Ekblom P Kemler R. Vasculogenesis and angiogenesis in embryonic-stem-cell-derived embryoid bodies. Development (1988) 102:471–8. 2460305 10.1242/dev.102.3.471

[4] Ricottini E Madonna R Grieco D Zoccoli A Stampachiacchiere B Patti G. Effect of high-dose atorvastatin reload on the release of endothelial progenitor cells in patients on long-term statin treatment who underwent percutaneous coronary intervention (from the ARMYDA-EPC Study). Am J Cardiol. (2016) 117:165–71. 10.1016/j.amjcard.2015.10.043 26743348

[5] Salazar-Martinez E Rodriguez-Valentin R Albavera-Hernandez C Carreon-Rodriguez A Lazcano-Ponce E. Number of colony-forming unit-Hill colonies among children and teenagers with obesity, dyslipidemia and breastfeeding history. Nutr Metab Cardiovasc Dis. (2016) 26:534–40. 10.1016/j.numecd.2016.03.010 27113291

[6] Hill JM Zalos G Halcox JP Schenke WH Waclawiw MA Quyyumi AA Finkel T. Circulating endothelial progenitor cells, vascular function, and cardiovascular risk. N Engl J Med. (2003) 348:593–600. 10.1056/NEJMoa022287 12584367

[7] Vaughan EE Liew A Mashayekhi K Dockery P McDermott J Kealy B. Pretreatment of endothelial progenitor cells with osteopontin enhances cell therapy for peripheral vascular disease. Cell Transplant. (2012) 21:1095–107. 10.3727/096368911X623880 22304991

[8] Reid E Guduric-Fuchs J O'Neill CL Allen LD Chambers SEJ Stitt AW. Preclinical evaluation and optimization of a cell therapy using human cord blood-derived endothelial colony-forming cells for ischemic retinopathies. Stem Cells Transl Med. (2018) 7:59–67. 10.1002/sctm.17-0187 29164803 PMC5746158

[9] Liew A Barry F O'Brien T. Endothelial progenitor cells: diagnostic and therapeutic considerations. Bioessays (2006) 28:261–70. 10.1002/bies.20372 16479582

[10] Medina RJ Barber CL Sabatier F Dignat-George F Melero-Martin JM Khosrotehrani K. Endothelial progenitors: a consensus statement on nomenclature. Stem Cells Transl Med. (2017) 6:1316–20. 10.1002/sctm.16-0360 28296182 PMC5442722

[11] Ingram DA Mead LE Tanaka H Meade V Fenoglio A Mortell K. Identification of a novel hierarchy of endothelial progenitor cells using human peripheral and umbilical cord blood. Blood (2004) 104:2752–60. 10.1182/blood-2004-04-1396 15226175

[12] Medina RJ O'Neill CL Sweeney M Guduric-Fuchs J Gardiner TA Simpson DA. Molecular analysis of endothelial progenitor cell (EPC) subtypes reveals two distinct cell populations with different identities. BMC Med Genomics (2010) 3:18. 10.1186/1755-8794-3-18 20465783 PMC2881111

[13] Sieveking DP Buckle A Celermajer DS Ng MK. Strikingly different angiogenic properties of endothelial progenitor cell subpopulations: insights from a novel human angiogenesis assay. J Am Coll Cardiol. (2008) 51:660–8. 10.1016/j.jacc.2007.09.059 18261686

[14] Hur J Yoon CH Kim HS Choi JH Kang HJ Hwang KK. Characterization of two types of endothelial progenitor cells and their different contributions to neovasculogenesis. Arterioscler Thromb Vasc Biol. (2004) 24:288–93. 10.1161/01.ATV.0000114236.77009.06 14699017

[15] Hirschi KK Ingram DA Yoder MC. Assessing identity, phenotype, and fate of endothelial progenitor cells. Arterioscler Thromb Vasc Biol. (2008) 28:1584–95. 10.1161/ATVBAHA.107.155960 18669889 PMC5244813

[16] De Rosa S Seeger FH Honold J Fischer-Rasokat U Lehmann R Fichtlscherer S Procedural safety and predictors of acute outcome of intracoronary administration of progenitor cells in 775 consecutive procedures performed for acute myocardial infarction or chronic heart failure. Circ Cardiovasc Interv. (2013) 6:44–51. 10.1161/CIRCINTERVENTIONS.112.971705 23362308

[17] Jimenez-Quevedo P Gonzalez-Ferrer JJ Sabate M Garcia-Moll X Delgado-Bolton R Llorente L. Selected CD133(+) progenitor cells to promote angiogenesis in patients with refractory angina: final results of the PROGENITOR randomized trial. Circ Res. (2014) 115:950–60. 10.1161/CIRCRESAHA.115.303463 25231095

[18] Arici V Perotti C Fabrizio C Del Fante C Ragni F Alessandrino F. Autologous immuno magnetically selected CD133+ stem cells in the treatment of no-option critical limb ischemia: clinical and contrast enhanced ultrasound assessed results in eight patients. J Transl Med. (2015) 13:342. 10.1186/s12967-015-0697-4 26526721 PMC4630831

[19] Mutirangura P Ruangsetakit C Wongwanit C Chinsakchai K Porat Y Belleli A. Enhancing limb salvage by non-mobilized peripheral blood angiogenic cell precursors therapy in patients with critical limb ischemia. J Med Assoc Thai. (2009) 92:320-7. Available online at: https://pdfs.semanticscholar.org/83a1/d9c5280b6ce4781840e794fc3c10499f7a58.pdf 19301723

[20] Zhang X Lian W Lou W Han S Lu C Zuo K. Transcatheter arterial infusion of autologous CD133(+) cells for diabetic peripheral artery disease. Stem Cells Int. (2016) 2016:6925357. 10.1155/2016/6925357 26981134 PMC4769775

[21] Kawamoto A Katayama M Handa N Kinoshita M Takano H Horii M. Intramuscular transplantation of G-CSF-mobilized CD34(+) cells in patients with critical limb ischemia: a phase I/IIa, multicenter, single-blinded, dose-escalation clinical trial. Stem Cells (2009) 27:2857–64. 10.1002/stem.207 19711453

[22] Kinoshita M Fujita Y Katayama M Baba R Shibakawa M Yoshikawa K. Long-term clinical outcome after intramuscular transplantation of granulocyte colony stimulating factor-mobilized CD34 positive cells in patients with critical limb ischemia. Atherosclerosis (2012) 224:440–5. 10.1016/j.atherosclerosis.2012.07.031 22877866

[23] Huang P Li S Han M Xiao Z Yang R Han ZC. Autologous transplantation of granulocyte colony-stimulating factor-mobilized peripheral blood mononuclear cells improves critical limb ischemia in diabetes. Diabetes Care (2005) 28:2155–60. 10.2337/diacare.28.9.2155 16123483

[24] Maione C Botti C Coppola CA Silvestroni C Lillo S Schiavone V. Effect of autologous transplantation of bone marrow cells concentrated with the MarrowXpress system in patients with critical limb ischemia. Transplant Proc. (2013) 45:402–6. 10.1016/j.transproceed.2012.10.031 23375329

[25] Cobellis G Botti C Taddeo A Silvestroni A Lillo S Da Ponte A. Successful bone marrow transplantation reveals the lack of endothelial progenitor cells mobilization in a patient with critical limb ischemia: a case report. Transplant Proc. (2010) 42:2816–20. 10.1016/j.transproceed.2010.04.047 20832596

[26] Wang XX Zhang FR Shang YP Zhu JH Xie XD Tao QM. Transplantation of autologous endothelial progenitor cells may be beneficial in patients with idiopathic pulmonary arterial hypertension: a pilot randomized controlled trial. J Am Coll Cardiol. (2007) 49:1566–71. 10.1016/j.jacc.2006.12.037 17418297

[27] Granton J Langleben D Kutryk MB Camack N Galipeau J Courtman DW Stewart DJ. Endothelial NO-synthase gene-enhanced progenitor cell therapy for pulmonary arterial hypertension: the PHACeT trial. Circ Res. (2015) 117:645–54. 10.1161/CIRCRESAHA.114.305951 26195220

[28] D'Avola D Fernandez-Ruiz V Carmona-Torre F Mendez M Perez-Calvo J Prosper F. Phase 1-2 pilot clinical trial in patients with decompensated liver cirrhosis treated with bone marrow-derived endothelial progenitor cells. Transl Res. (2017) 188:80–91e2. 10.1016/j.trsl.2016.02.009 26972567

[29] Maldonado GE Perez CA Covarrubias EE Cabriales SA Leyva LA Perez JC. Autologous stem cells for the treatment of post-mastectomy lymphedema: a pilot study. Cytotherapy (2011) 13:1249–55. 10.3109/14653249.2011.594791 21999374

[30] Vasyliev RG Oksymets VM Rodnichenko AE Zlatska AV Gubar OS Gordiienko IM. Tissue-engineered bone for treatment of combat related limb injuries. Exp Oncol. (2017) 39:191–6. Available online at: http://exp-oncology.com.ua/article/10281 28967639

[31] Lara-Hernandez R Lozano-Vilardell P Blanes P Torreguitart-Mirada N Galmes A Besalduch J. Safety and efficacy of therapeutic angiogenesis as a novel treatment in patients with critical limb ischemia. Ann Vasc Surg. (2010) 24:287–94. 10.1016/j.avsg.2009.10.012 20142004

[32] Tanaka R Masuda H Kato S Imagawa K Kanabuchi K Nakashioya C. Autologous G-CSF-mobilized peripheral blood CD34+ cell therapy for diabetic patients with chronic nonhealing ulcer. Cell Transplant. (2014) 23:167–79. 10.3727/096368912X658007 23107450

[33] Zhu J Song J Yu L Zheng H Zhou B Weng S Fu G. Safety and efficacy of autologous thymosin beta4 pre-treated endothelial progenitor cell transplantation in patients with acute ST segment elevation myocardial infarction: a pilot study. Cytotherapy (2016) 18:1037–42. 10.1016/j.jcyt.2016.05.006 27288307

[34] Zhu JH Wang XX Zhang FR Shang YP Tao QM Zhu JH. Safety and efficacy of autologous endothelial progenitor cells transplantation in children with idiopathic pulmonary arterial hypertension: open-label pilot study. Pediatr Transplant. (2008) 12:650–5. 10.1111/j.1399-3046.2007.00863.x 18466198

[35] Bennis Y Sarlon-Bartoli G Guillet B Lucas L Pellegrini L Velly L. Priming of late endothelial progenitor cells with erythropoietin before transplantation requires the CD131 receptor subunit and enhances their angiogenic potential. J Thromb Haemost. (2012) 10:1914–28. 10.1111/j.1538-7836.2012.04835.x 22738133

[36] Sarlon G Zemani F David L Duong Van Huyen JP Dizier B Grelac F. Therapeutic effect of fucoidan-stimulated endothelial colony-forming cells in peripheral ischemia. J Thromb Haemost. (2012) 10:38–48. 10.1111/j.1538-7836.2011.04554.x 22066680

[37] Flex A Biscetti F Iachininoto MG Nuzzolo ER Orlando N Capodimonti S. Human cord blood endothelial progenitors promote post-ischemic angiogenesis in immunocompetent mouse model. Thromb Res. (2016) 141:106–11. 10.1016/j.thromres.2016.03.012 26994683

[38] Mena HA Lokajczyk A Dizier B Strier SE Voto LS Boisson-Vidal C. Acidic preconditioning improves the proangiogenic responses of endothelial colony forming cells. Angiogenesis (2014) 17:867–79. 10.1007/s10456-014-9434-5 24854678

[39] Kim BR Jang IH Shin SH Kwon YW Heo SC Choi EJ. Therapeutic angiogenesis in a murine model of limb ischemia by recombinant periostin and its fasciclin I domain. Biochim Biophys Acta. (2014) 1842:1324–32. 10.1016/j.bbadis.2014.05.004 24834847

[40] Goto K Takemura G Takahashi T Okada H Kanamori H Kawamura I. Intravenous administration of endothelial colony-forming cells overexpressing integrin beta1 augments angiogenesis in ischemic legs. Stem Cells Transl Med. (2016) 5:218–26. 10.5966/sctm.2015-0096 26702126 PMC4729550

[41] Odent Grigorescu G Preda MB Radu E Rosca AM Tutuianu R Mitroi DN Combinatorial approach for improving the outcome of angiogenic therapy in ischemic tissues. Biomaterials (2015) 60:72–81. 10.1016/j.biomaterials.2015.05.002 25985154

[42] Lee JH Lee SH Yoo SY Asahara T Kwon SM. CD34 hybrid cells promote endothelial colony-forming cell bioactivity and therapeutic potential for ischemic diseases. Arterioscler Thromb Vasc Biol. (2013) 33:1622–34. 10.1161/ATVBAHA.112.301052 23640491

[43] Schwarz TM Leicht SF Radic T Rodriguez-Arabaolaza I Hermann PC Berger F. Vascular incorporation of endothelial colony-forming cells is essential for functional recovery of murine ischemic tissue following cell therapy. Arterioscler Thromb Vasc Biol. (2012) 32:e13–21. 10.1161/ATVBAHA.111.239822 22199368

[44] Mena HA Zubiry PR Dizier B Schattner M Boisson-Vidal C Negrotto S. Acidic preconditioning of endothelial colony-forming cells (ECFC) promote vasculogenesis under proinflammatory and high glucose conditions *in vitro* and *in vivo*. Stem Cell Res Ther. (2018) 9:120. 10.1186/s13287-018-0872-7 29720269 PMC5930427

[45] d'Audigier C Susen S Blandinieres A Mattot V Saubamea B Rossi E. Egfl7 Represses the vasculogenic potential of human endothelial progenitor cells. Stem Cell Rev. (2018) 14:82–91. 10.1007/s12015-017-9775-8 28980146

[46] Fraineau S Palii CG McNeill B Ritso M Shelley WC Prasain N. Epigenetic activation of pro-angiogenic signaling pathways in human endothelial progenitors increases vasculogenesis. Stem Cell Rep. (2017) 9:1573–87. 10.1016/j.stemcr.2017.09.009 29033304 PMC5830028

[47] Poitevin S Cussac D Leroyer AS Albinet V Sarlon-Bartoli G Guillet B. Sphingosine kinase 1 expressed by endothelial colony-forming cells has a critical role in their revascularization activity. Cardiovasc Res. (2014) 103:121–30. 10.1093/cvr/cvu104 24743591

[48] Palii CG Vulesevic B Fraineau S Pranckeviciene E Griffith AJ Chu A. Trichostatin A enhances vascular repair by injected human endothelial progenitors through increasing the expression of TAL1-dependent genes. Cell Stem Cell (2014) 14:644–57. 10.1016/j.stem.2014.03.003 24792117

[49] Heo SC Kwon YW Jang IH Jeong GO Yoon JW Kim CD. WKYMVm-induced activation of formyl peptide receptor 2 stimulates ischemic neovasculogenesis by promoting homing of endothelial colony-forming cells. Stem Cells (2014) 32:779–90. 10.1002/stem.1578 24155208

[50] Bouvard C Gafsou B Dizier B Galy-Fauroux I Lokajczyk A Boisson-Vidal C. alpha6-integrin subunit plays a major role in the proangiogenic properties of endothelial progenitor cells. Arterioscler Thromb Vasc Biol. (2010) 30:1569–75. 10.1161/ATVBAHA.110.209163 20508204

[51] Smadja DM Bieche I Silvestre JS Germain S Cornet A Laurendeau I. Bone morphogenetic proteins 2 and 4 are selectively expressed by late outgrowth endothelial progenitor cells and promote neoangiogenesis. Arterioscler Thromb Vasc Biol. (2008) 28:2137–43. 10.1161/ATVBAHA.108.168815 18818419

[52] Cubbon RM Yuldasheva NY Viswambharan H Mercer BN Baliga V Stephen SL. Restoring Akt1 activity in outgrowth endothelial cells from South Asian men rescues vascular reparative potential. Stem Cells (2014) 32:2714–23. 10.1002/stem.1766 24916783

[53] Leicht SF Schwarz TM Hermann PC Seissler J Aicher A Heeschen C. Adiponectin pretreatment counteracts the detrimental effect of a diabetic environment on endothelial progenitors. Diabetes (2011) 60:652–61. 10.2337/db10-0240 21270275 PMC3028367

[54] Rossi E Smadja D Goyard C Cras A Dizier B Bacha N. Co-injection of mesenchymal stem cells with endothelial progenitor cells accelerates muscle recovery in hind limb ischemia through an endoglin-dependent mechanism. Thromb Haemost. (2017) 117:1908–18. 10.1160/TH17-01-0007 28771278

[55] Medina RJ O'Neill CL O'Doherty TM Chambers SE Guduric-Fuchs J Neisen J. *Ex vivo* expansion of human outgrowth endothelial cells leads to IL-8-mediated replicative senescence and impaired vasoreparative function. Stem Cells (2013) 31:1657–68. 10.1002/stem.1414 23629812

[56] Tan Q Qiu L Li G Li C Zheng C Meng H Transplantation of healthy but not diabetic outgrowth endothelial cells could rescue ischemic myocardium in diabetic rabbits. Scand J Clin Lab Invest. (2010) 70:313–21. 10.3109/00365511003774593 20470214

[57] Kim SW Jin HL Kang SM Kim S Yoo KJ Jang Y. Therapeutic effects of late outgrowth endothelial progenitor cells or mesenchymal stem cells derived from human umbilical cord blood on infarct repair. Int J Cardiol. (2016) 203:498–507. 10.1016/j.ijcard.2015.10.110 26551883 PMC4688096

[58] Kang KT Coggins M Xiao C Rosenzweig A Bischoff J. Human vasculogenic cells form functional blood vessels and mitigate adverse remodeling after ischemia reperfusion injury in rats. Angiogenesis (2013) 16:773–84. 10.1007/s10456-013-9354-9 23666122 PMC3773287

[59] Burger D Vinas JL Akbari S Dehak H Knoll W Gutsol A. Human endothelial colony-forming cells protect against acute kidney injury: role of exosomes. Am J Pathol. (2015) 185:2309–23. 10.1016/j.ajpath.2015.04.010 26073035

[60] Wang SH Lin SJ Chen YH Lin FY Shih JC Wu CC. Late outgrowth endothelial cells derived from Wharton jelly in human umbilical cord reduce neointimal formation after vascular injury: involvement of pigment epithelium-derived factor. Arterioscler Thromb Vasc Biol. (2009) 29:816–22. 10.1161/ATVBAHA.109.184739 19342598

[61] Xia WH Chen L Liang JW Zhang XY Su C Tong X. BMP4/Id2 signaling pathway is a novel therapeutic target for late outgrowth endothelial progenitor cell-mediated endothelial injury repair. Int J Cardiol. (2017) 228:796–804. 10.1016/j.ijcard.2016.11.027 27888757

[62] Huang XT Zhang YQ Li SJ Li SH Tang Q Wang ZT. Intracerebroventricular transplantation of *ex vivo* expanded endothelial colony-forming cells restores blood-brain barrier integrity and promotes angiogenesis of mice with traumatic brain injury. J Neurotrauma. (2013) 30:2080–8. 10.1089/neu.2013.2996 23957220 PMC3868401

[63] Zhang Y Li Y Wang S Han Z Huang X Li S. Transplantation of expanded endothelial colony-forming cells improved outcomes of traumatic brain injury in a mouse model. J Surg Res. (2013) 185:441–9. 10.1016/j.jss.2013.05.073 23953790

[64] Garrigue P Hache G Bennis Y Brige P Stalin J Pellegrini L. Erythropoietin pretreatment of transplanted endothelial colony-forming cells enhances recovery in a cerebral ischemia model by increasing their homing ability: a SPECT/CT study. J Nucl Med. (2016) 57:1798–804. 10.2967/jnumed.115.170308 27609786

[65] Pellegrini L Bennis Y Guillet B Velly L Garrigue P Sabatier F. Therapeutic benefit of a combined strategy using erythropoietin and endothelial progenitor cells after transient focal cerebral ischemia in rats. Neurol Res. (2013) 35:937–47. 10.1179/1743132813Y.0000000235 23816235

[66] Moubarik C Guillet B Youssef B Codaccioni JL Piercecchi MD Sabatier F. Transplanted late outgrowth endothelial progenitor cells as cell therapy product for stroke. Stem Cell Rev. (2011) 7:208–20. 10.1007/s12015-010-9157-y 20526754

[67] Ding J Zhao Z Wang C Wang CX Li PC Qian C. Bioluminescence imaging of transplanted human endothelial colony-forming cells in an ischemic mouse model. Brain Res. (2016) 1642:209–18. 10.1016/j.brainres.2016.03.045 27038754

[68] Coppens V Heremans Y Leuckx G Suenens K Jacobs-Tulleneers-Thevissen D Verdonck K. Human blood outgrowth endothelial cells improve islet survival and function when co-transplanted in a mouse model of diabetes. Diabetologia (2013) 56:382–90. 10.1007/s00125-012-2754-3 23090187

[69] Kim JH Oh BJ Lee HN Park HS Park SG Park KS. Endothelial colony-forming cell coating of pig islets prevents xenogeneic instant blood-mediated inflammatory reaction. Cell Transplant. (2011) 20:1805–15. 10.3727/096368911X566154 21396165

[70] Jung HS Kim MJ Hong SH Lee YJ Kang S Lee H. The potential of endothelial colony-forming cells to improve early graft loss after intraportal islet transplantation. Cell Transplant. (2014) 23:273–83. 10.3727/096368912X661364 23294520

[71] Ormiston ML Deng Y Stewart DJ Courtman DW. Innate immunity in the therapeutic actions of endothelial progenitor cells in pulmonary hypertension. Am J Respir Cell Mol Biol. (2010) 43:546–54. 10.1165/rcmb.2009-0152OC 19995942

[72] Alphonse RS Vadivel A Fung M Shelley WC Critser PJ Ionescu L. Existence, functional impairment, and lung repair potential of endothelial colony-forming cells in oxygen-induced arrested alveolar growth. Circulation (2014) 129:2144–57. 10.1161/CIRCULATIONAHA.114.009124 24710033 PMC4162516

[73] Aide N Desmonts C Beauregard JM Beyer T Kinross K Roselt P. High throughput static and dynamic small animal imaging using clinical PET/CT: potential preclinical applications. Eur J Nucl Med Mol Imaging (2010) 37:991–1001. 10.1007/s00259-009-1352-1 20107792

[74] Martin-Ramirez J Hofman M van den Biggelaar M Hebbel RP Voorberg J. Establishment of outgrowth endothelial cells from peripheral blood. Nat Protoc. (2012) 7:1709–15. 10.1038/nprot.2012.093 22918388

[75] Liew A O'Brien T Isolation of Endothelial Progenitor Cells (EPCs). In: Slevin M McDowell G editors. Handbook of Vascular Biology Techniques. Dordrecht: Springer Netherlands (2015). p. 45–54.

[76] Mead LE Prater D Yoder MC Ingram DA. Isolation and characterization of endothelial progenitor cells from human blood. Curr Protoc Stem Cell Biol. (2008) Chapter 2:Unit 2C 1. 10.1002/9780470151808.sc02c01s6 18770637

[77] Hofmann NA Reinisch A Strunk D. Endothelial colony-forming progenitor cell isolation and expansion. Methods Mol Biol. (2012) 879:381–7. 10.1007/978-1-61779-815-3_23 22610572

[78] Alphonse RS Vadivel A Zhong S McConaghy S Ohls R Yoder MC Thebaud B. The isolation and culture of endothelial colony-forming cells from human and rat lungs. Nat Protoc. (2015) 10:1697–708. 10.1038/nprot.2015.107 26448359

[79] Colombo E Calcaterra F Cappelletti M Mavilio D Della Bella S. Comparison of fibronectin and collagen in supporting the isolation and expansion of endothelial progenitor cells from human adult peripheral blood. PLoS ONE (2013) 8:e66734. 10.1371/journal.pone.0066734 23824996 PMC3688932

[80] Tasev D van Wijhe MH Weijers EM van Hinsbergh VW Koolwijk P. Long-term expansion in platelet lysate increases growth of peripheral blood-derived endothelial-colony forming cells and their growth factor-induced sprouting capacity. PLoS ONE (2015) 10:e0129935. 10.1371/journal.pone.0129935 26076450 PMC4468160

[81] Reinisch A Hofmann NA Obenauf AC Kashofer K Rohde E Schallmoser K. Humanized large-scale expanded endothelial colony-forming cells function *in vitro* and *in vivo*. Blood (2009) 113:6716–25. 10.1182/blood-2008-09-181362 19321860 PMC2710924

[82] Siegel G Fleck E Elser S Hermanutz-Klein U Waidmann M Northoff H. Manufacture of endothelial colony-forming progenitor cells from steady-state peripheral blood leukapheresis using pooled human platelet lysate. Transfusion (2018) 58:1132–42. 10.1111/trf.14541 29473177

[83] Jarajapu YPR Hazra S Segal M LiCalzi S Jhadao C Qian K. Vasoreparative dysfunction of CD34+ cells in diabetic individuals involves hypoxic desensitization and impaired autocrine/paracrine mechanisms. PLOS ONE (2014) 9:e93965. 10.1371/journal.pone.0093965 24713821 PMC3979711

[84] Lin R-Z Dreyzin A Aamodt K Li D Jaminet S-CS Dudley AC. Induction of erythropoiesis using human vascular networks genetically engineered for controlled erythropoietin release. Blood (2011) 118:5420. 10.1182/blood-2011-08-372946 21937702 PMC3217346

[85] Choi JH Hur J Yoon CH Kim JH Lee CS Youn SW. Augmentation of therapeutic angiogenesis using genetically modified human endothelial progenitor cells with altered glycogen synthase kinase-3beta activity. J Biolog Chem. (2004) 279:49430–8. 10.1074/jbc.M402088200 15339925

[86] Souidi N Stolk M Rudeck J Strunk D Schallmoser K Volk HD. Stromal cells act as guardians for endothelial progenitors by reducing their immunogenicity after co-transplantation. Stem Cells (2017) 35:1233–45. 10.1002/stem.2573 28100035

[87] Allen P Kang KT Bischoff J. Rapid onset of perfused blood vessels after implantation of ECFCs and MPCs in collagen, PuraMatrix and fibrin provisional matrices. J Tissue Eng Regen Med. (2015) 9:632–6. 10.1002/term.1803 23955835

[88] Kang KT Lin RZ Kuppermann D Melero-Martin JM Bischoff J. Endothelial colony forming cells and mesenchymal progenitor cells form blood vessels and increase blood flow in ischemic muscle. Sci Rep. (2017) 7:770. 10.1038/s41598-017-00809-1 28396600 PMC5429692

[89] Lin RZ Moreno-Luna R Zhou B Pu WT Melero-Martin JM. Equal modulation of endothelial cell function by four distinct tissue-specific mesenchymal stem cells. Angiogenesis (2012) 15:443–55. 10.1007/s10456-012-9272-2 22527199 PMC3409933

[90] Shafiee A Patel J Wong HY Donovan P Hutmacher DW Fisk NM. Priming of endothelial colony-forming cells in a mesenchymal niche improves engraftment and vasculogenic potential by initiating mesenchymal transition orchestrated by NOTCH signaling. FASEB J. (2017) 31:610–24. 10.1096/fj.201600937 28045376

[91] Moher D Liberati A Tetzlaff J Altman DG The PG Preferred reporting items for systematic reviews and meta-analyses: the PRISMA statement. PLOS Med. (2009) 6:e1000097 10.1371/journal.pmed.1000097 19621072 PMC2707599

