# Supplementary material for: Recent Advances in Endothelial Progenitor Cells Toward Their Use in Clinical Translation
Source: Front Med (Lausanne). 2018 Dec 18;5:354. doi: 10.3389/fmed.2018.00354 (PMC6305310; doi:10.3389/fmed.2018.00354)
Supplement: Supplementary file 1 [file Table_1.pdf]

Supplementary Table 1: Published studies using EPCs as a therapeutic agent.

Condition	Treatment	Source	Culture Conditions	Cell Type	n	Outcome	Reference
Liver Cirrhosis	Single injection of autologous EPCs into the hepatic artery	BM	Cultured on fibronectin with endothelial complete medium	CD31, $CD34^+$, CD14, $VEGFR2^+$, $VEGFR1^+$, $CD133^+$, CD90, $CD117^+$, vWF^+, $CXCR4^+$, $CD45^+$, $ID1^+$	12	Treatment was safe and showed an improvement in liver function	D'Avola *et al*[21]
CLI	Autologous EPC injected IM into the ischemic site	PB	Cells were injected 24hrs after apheresis without any culture	$CD34^+$, $CD133^+$	28	Decreased pain scores and increased amputation-free rate.	Lara-Hernandez *et al*[17]
Non-Healing Diabetic Ulcers	Autologous EPCs were injected IM within 20 cm surrounding the wound	PB	Cells were injected on the same day as isolation with no culture	$CD34^+$	5	Increased vascular perfusion and wound closure & safety	Tanaka *et al*[18]
STEMI	Autologous thymosin β4 treated EPCs were injected into distal part of IRA	PB	Cultured on fibronectin with Medium-199 + 20% patient serum + 50ng/ml VEGF	NR	10	Increased exercise function and left ventricular function & safety	Zhu *et al*[19]
Idiopathic PAH	IV infusion of autologous EPCs	PB	Cultured on fibronectin with Medium-199 + 20% patient serum + 50ng/ml VEGF	NR	13	Significant increase in exercise capacity and pulmonary haemodynamics	Zhu *et al*[20]
PAH	eNOS-EPCs injected into the right atrium via a catheter	PB	Cultured on fibronectin	$CD14^+$, $CD31^+$	7	Significant improvement in 6 minute walk test after 3 months	Granton *et al*[32]
Traumatic Bone Defect	EPCs in a 3-dimensional tissue engineered bone equivalent scaffold	PB	Cultured in EGM-2 in uncoated flasks	$CD105^+$, $CD90^-$, $CD73^+$, $CD271^-$, $CD31^+$, $CD34^+$, $CD45^-$, HLA-DR^-	20	Bone restoration after 5-6 months	Vasyliev *et al*[33]

BM = Bone Marrow, CLI = Critical Limb Ischaemia, eNOS = Endothelial Nitric Oxide Synthase, EPC = Endothelial Progenitor Cell, IM = Intramuscular, PAH = Pulmonary Arterial Hypertension, IRA = Infarct Related Artery, IV = Intravenous, PB = Peripheral Blood, STEMI = ST Segment Elevated Myocardial Infarction, VEGFR = Vascular Endothelial Growth Factor Receptor, vWF = Von Willebrands Factor.
