# Supplementary material for: Recent Advances in Endothelial Progenitor Cells Toward Their Use in Clinical Translation
Source: Front Med (Lausanne). 2018 Dec 18;5:354. doi: 10.3389/fmed.2018.00354 (PMC6305310; doi:10.3389/fmed.2018.00354)
Supplement: Supplementary file 2 [file Data_Sheet_1.pdf]

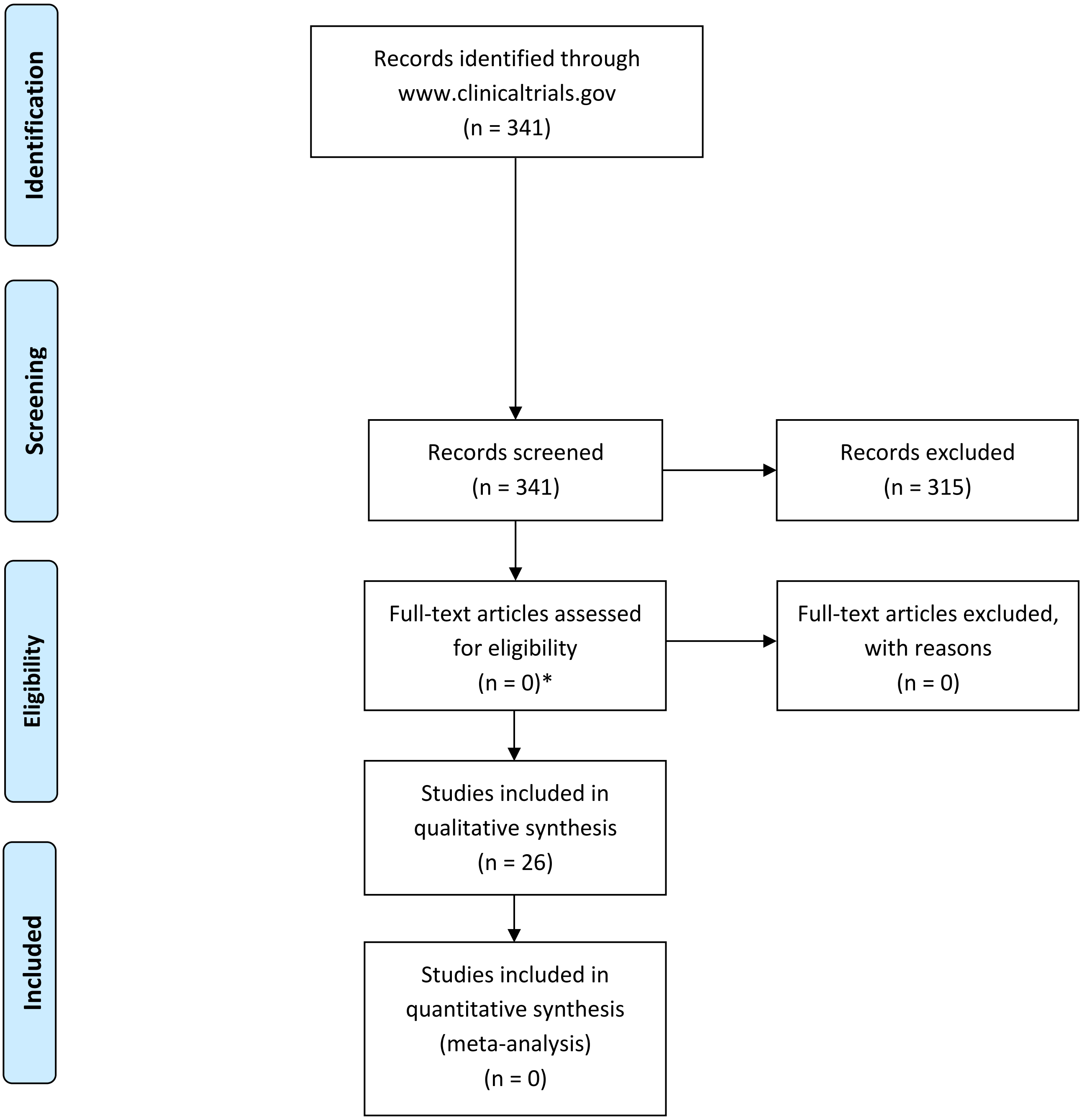

*There was no published articles within the www.clinicaltrials.gov database

Appendix 1: PRISMA Flow diagram (Adapted from Moher *et al*[58]) for clinical trials using EPCs as therapeutic agent, registered under www.clinicalrtrials.gov registry.

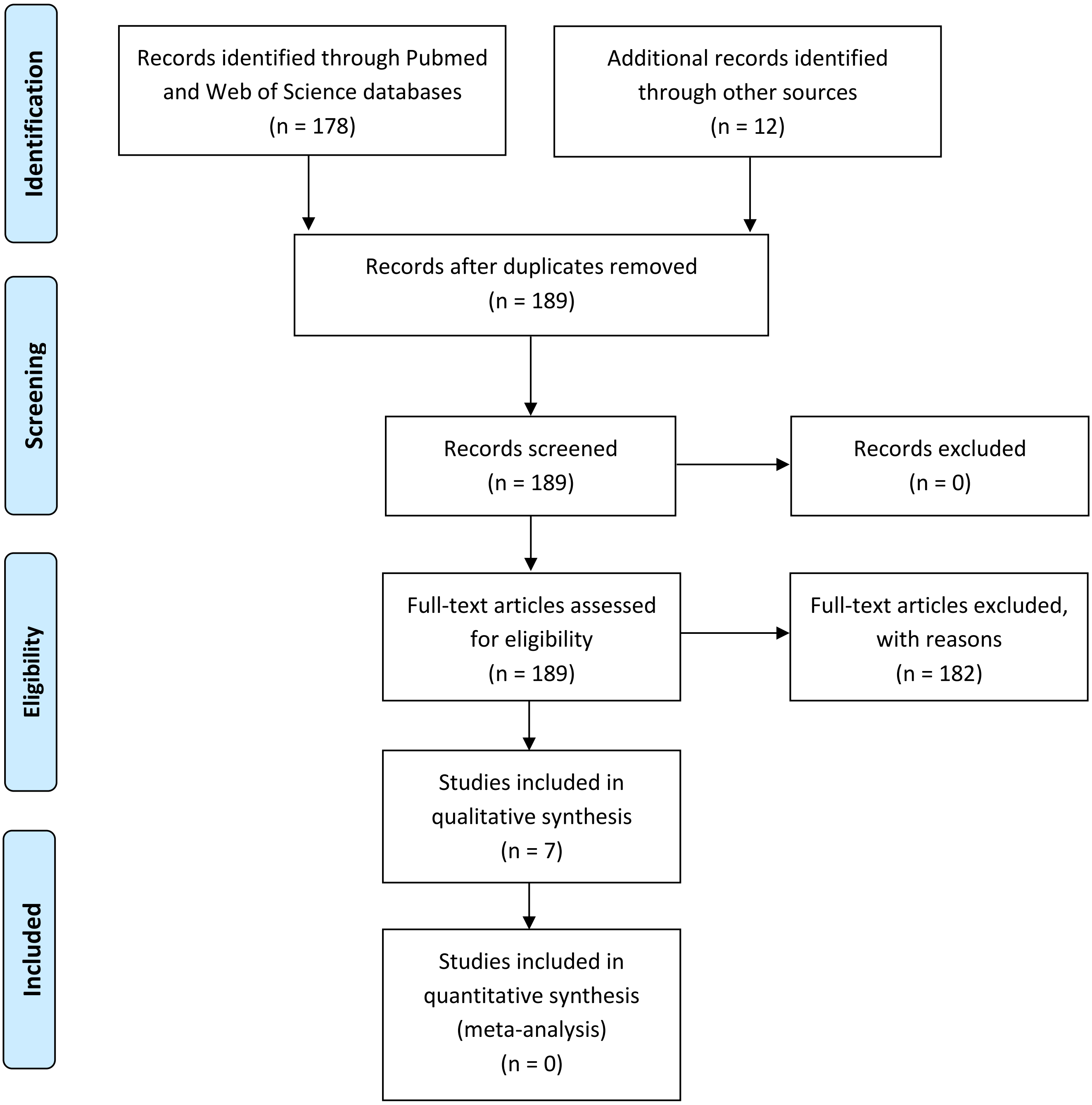

Appendix 2: PRISMA Flow diagram (Adapted from Moher *et al*[58]) for published studies using EPCs as a therapeutic agent.

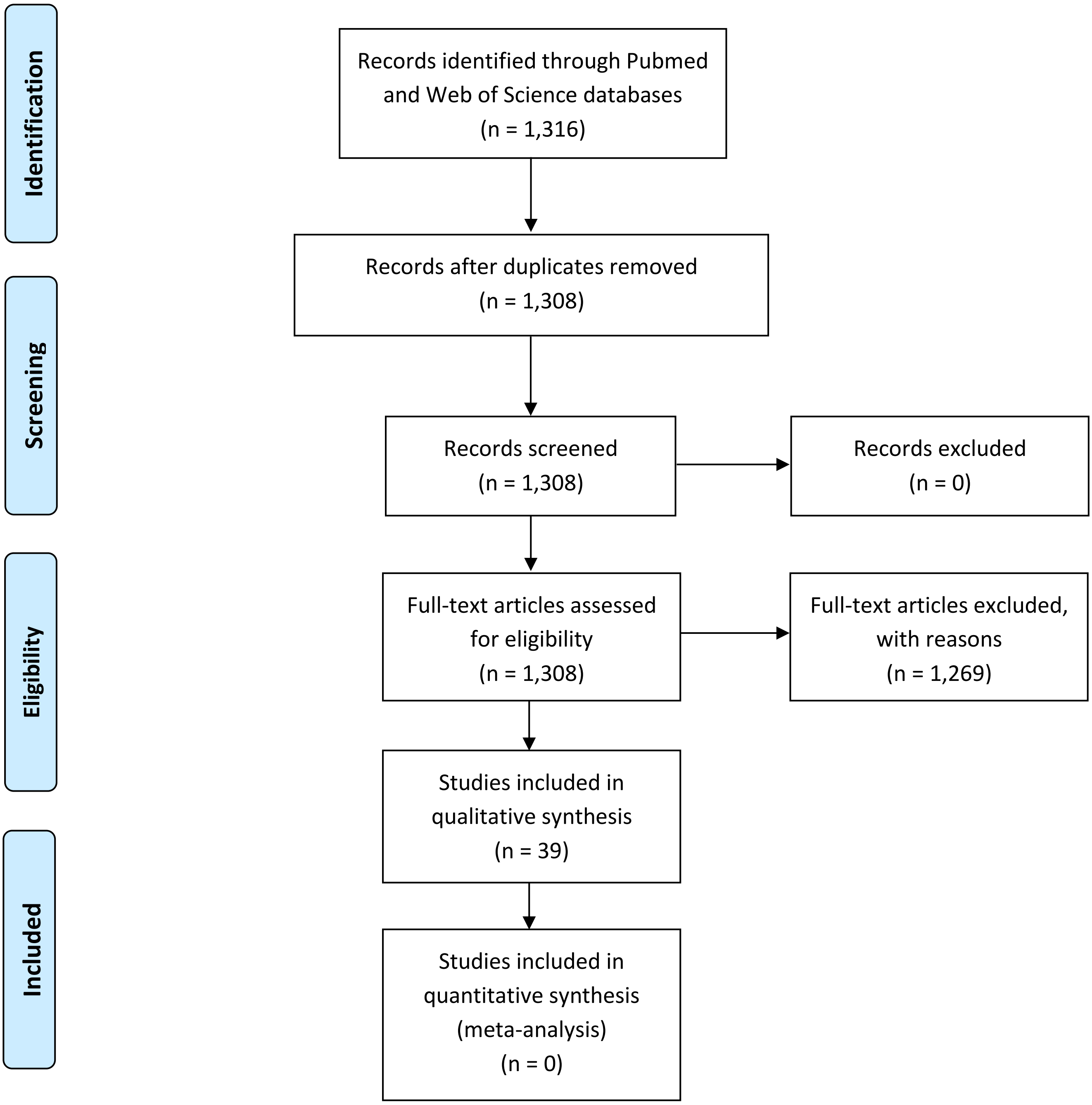

Appendix 3: PRISMA Flow diagram (Adapted from Moher *et al*[58]) for preclinical studies using ECFCs as a therapeutic agent.

.
